# Cannula Implantation Reduces the Severity of the Beta Amyloid Effect on Peroxidized Lipids and Glutathione Levels in the Brain of BALB/c Mice

**DOI:** 10.32607/actanaturae.27439

**Published:** 2024

**Authors:** K. A. Mukhina, V. A. Mitkevich, I. Yu. Popova

**Affiliations:** Engelhardt Institute of Molecular Biology, Russian Academy of Sciences, Moscow, 119991 Russian Federation; Institute of Theoretical and Experimental Biophysics, Russian Academy of Sciences, Pushchino, 142290 Russian Federation

**Keywords:** amyloid toxicity, immunohistochemistry, microglia, reactive oxygen species, glutathione, lipid peroxides

## Abstract

Sporadic Alzheimer’s disease (sAD) is the most common of neurodegenerative disorders. The lack of effective therapy indicates that the mechanisms of sAD development remain poorly understood. To investigate this pathology in animals, intracerebroventricular injection of β-amyloid peptide (Aβ) using a Hamilton syringe, either during stereotactic surgery or through a pre-implanted cannula, is used. In this study, we analyzed the effect of chronic cannula implantation on the severity of Aβ effects at the behavioral, histological, and biochemical levels. The results showed that the local damage to neural tissue caused by cannulation has no bearing on the effect of Aβ on animal behavior and the microglial parameters of the unilateral hippocampus two weeks after the Aβ administration. However, cannula implantation fundamentally modifies some biochemical markers of the oxidative stress that occurs in the brain tissue in response to Aβ administration. Thus, the presence of a cannula reduces the severity of the Aβ impact on the levels of peroxidized lipids and glutathione two- and 10-fold, respectively. It is important to note that the detected changes are chronic and systemic. This is known because the homogenate of the entire contralateral (in relation to the cannula implantation site) hemisphere was analyzed, and the analysis was performed two weeks after implantation. At the same time, cannulation does not affect the rate of reactive oxygen species production. The obtained data indicate that chronic implantation of a cannula into the brain of experimental animals fundamentally distorts some parameters of oxidative stress in the neural tissue, which are widely used to assess the severity of experimental Alzheimer’s-type diseases.

## INTRODUCTION

One of the central problems in modern neurobiology is developing an effective therapy for sporadic (non-hereditary) Alzheimer’s disease (sAD). To date, sAD, which accounts for up to 95% of all diagnosed Alzheimer’s disease (AD) cases, is the third most common cause of death after cerebrovascular diseases and cancer [1]. On current estimates, the number of people diagnosed with sAD will double every 20 years, reaching 66 × 10^6^ and 115 × 10^6^ people by 2030 and 2050, respectively [2]. However, these estimates have not taken into consideration the COVID-19 pandemic outcomes, which might add significantly to the increase in the number of AD patients [3, 4]. It is believed that sAD is caused by a combination of genetic and environmental risk factors without a confirmed medical history of AD [5]. Many hypotheses on sAD etiology currently exist. The disease is associated with β-amyloid (Aβ) deposition [6], tau protein hyperphosphorylation [7], oxidative stress [8, 9], glucose hypometabolism [10], neuroinflammation [11], degeneration of cholinergic neurons [12], disruptions in the intestinal microbiota [13, 14], disruptions in the lipid metabolism [15], autophagy dysfunction [16], insulin resistance [17, 18], synaptic dysfunction [8, 19], etc. However, the lack of effective treatments indicates our insufficient understanding of sAD mechanisms. One of the common approaches used to study the mechanisms of sAD development is a single Aβ injection into the brain of sAD model animals [20, 21, 22]. Beta-amyloid is usually injected unilaterally, in the lateral ventricle using a Hamilton syringe during stereotaxic surgery or through a pre-implanted guide cannula. The cannula implant allows for direct, lengthy administration of various substances into the brain, including potential therapeutic drugs; however, this at the same time leads to additional damage to tissue, triggering a local inflammatory response [23]. This initial inflammatory response is mainly due to microglia activation, with the release of interleukin-1β [24, 25]. As shown in the control animals, the acute inflammatory response lasts less than one week [26]; past two weeks, glia alignment along the implanted object is observed [23]. Meanwhile, Aβ is known as a direct microglia activator [27, 28]. Thus, both chronic cannulation and Aβ act in the same direction. As a result, tissue response to cannula implantation may enhance or, conversely, mask the effect of Aβ. However, there is no experimental evidence in the literature to prove this. Yet it is obvious that distortions in the effects of Aβ in the study of sAD development mechanisms or the testing of potential therapeutic drugs may lead to false conclusions.

The aim of the current work was to study the effect of neural tissue damage resulting from cannula implantation on the formation of amyloid toxicity following intracerebroventricular (ICV) administration of Aβ to BALB/c mice. The presence and severity of the synergism of these effects was assessed at the systemic level using behavioral tests and at the cellular and subcellular levels using immunohistochemical and biochemical methods two weeks after Aβ administration.

## EXPERIMENTAL

Experiments were conducted in male BALB/c mice weighing 25–33 g. Mice were received from the Stolbovaya Breeding Nursery (https://www.pitst.ru/, Moscow Region, Chekhov District, Stolbovaya settlement). The animals were housed in individual cages with a 12-hour light/dark cycle and access to food and water *ad libitum *in a room with controlled temperature (22°C). All studies were conducted in accordance with the Ethical Principles for Biomedical Research of the 1964 Helsinki Declaration and approved by the Biosafety and Bioethics Committee of the Institute of Theoretical and Experimental Biophysics of the Russian Academy of Sciences (Pushchino), protocol No. 40/2023 dated February 15, 2023.

**Fig. 1:**
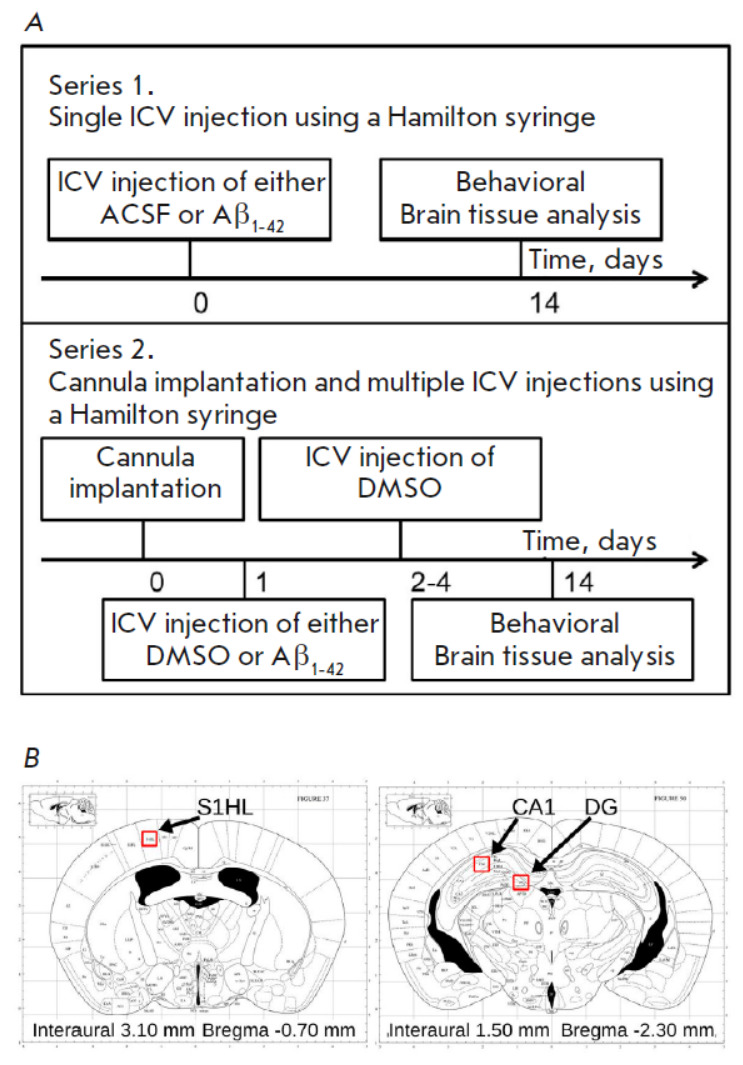
(*A*) Schematic representation of the experiment. (*B*) Atlas image of the frontal section of the mouse brain [29] with indication of the site of a guide cannula implantation (left) and CA1 and dentate gyrus areas for microglia cell quantification (right)

**The experimental scheme** is presented in *Fig. 1A*. Two series of experiments were conducted. The animals in the first series underwent neurosurgery. During the surgery, they were injected either 1 μl of an artificial cerebrospinal fluid (ACSF) (group C) or 1 μl of Aβ1–42 to model sAD (group AD) in the left lateral ventricle of the animal brain using a Hamilton syringe. The animals in the second group underwent neurosurgery with implantation of a guide cannula for drug administration. The drugs were administered through the cannula using a Hamilton syringe 24 h after the surgery. Since the cannula is implanted for repeated drug administration, and the drugs are often dissolved in DMSO, we replaced the ACSF solution with DMSO in this experimental series. On the first day of administration, the first group of animals received 1 μl of DMSO (group C-C) and the other group received 1 μl of Aβ1–42+DMSO (group AD-C). All animals continued to receive DMSO for the following 3 days.

The behavioral open field test was performed 14 days after Aβ1–42 administration. Next, the mice were anesthetized with isoflurane. The brain was removed. Half of the brain was fixed in paraformaldehyde for a immunohistochemical analysis, and the other half was analyzed biochemically.

**Stereotaxic surgeries **were performed under general gaseous anesthesia using isoflurane (1–4%; oxygen partial pressure, 0.8). The coordinates for Hamilton syringe implantation (G29) were identical for all the groups of animals: AP = -0.7; L = 1.4; h = 2.2 [29]. The guide cannula (stainless steel; size G21) for microinjections was positioned above the left lateral ventricle at the following coordinates: AP = -0.7; L = 1.4; h = 1.5 [29] (*Fig. 1B*).

**Drug microinjections**

The control animals received either ACSF (NaCl 0.9% + PBS + 5 mM glucose) or DMSO (Sigma-Aldrich, USA). The AD groups were injected with Aβ1–42 (Sigma-Aldrich). Oligomeric Aβ1–42 (1 mg, Sigma- Aldrich) was dissolved in 1 ml of a 1.0% NH_4_OH solution in saline and sonicated for 1 min. Aliquots were stored at –80°C. Prior to the experiments, the Aβ1–42 solution was fibrillized for 24 h at 36°C and sonicated for 1 min. Solutions were injected at a rate of 1 μl/min in a volume of 1 μl.

**Behavior**

To assess the general condition of the animals, we used the Open Field test. The test represents a brightly illuminated open black circular arena 60 cm in diameter and with a wall height of 50 cm. The behavior of each animal was recorded individually for 3 min using a video camera. The EthoVision software (Noldus Information Technology, the Netherlands) was used for video recording and analysis. Behavior was analyzed in two zones: in the center (d = 20 cm) and along the walls. The following parameters were assessed: time spent in each zone, movement/immobility ratio in each zone, frequency in entering the central zone, the latency period before the first entrance in the zone along the walls, the total distance traveled, and the average speed of movement. The Open Field setup was sanitized with a 10% alcohol solution for each animal.

**Histology**

The brain’s left hemisphere was placed in cold paraformaldehyde for fixation and storage. Sections were made onto a Leica VT 1200 S vibratome (Leica, Germany). Frontal 35-μm sections were obtained, and microglia were stained with primary rabbit anti- Iba-1 antibodies (1 : 1 000; Wako, Japan) according to the manufacturer’s instructions. Next, secondary goat anti-rabbit antibodies (1 : 1 000; Alexa Fluor 488, ThermoFisher, USA) were used. After staining, the sections were mounted on slides and microglia fluorescence was analyzed on a Nikon E200 microscope at ×40 magnification. The number and total area of Iba-1+ cells in 300 × 300 μm squares were calculated. The analysis was performed in the CA1 area and the dentate gyrus (DG) of the hippocampus (*Fig. 1B*) using ImageJ software (NIH, USA); six sections from each brain were analyzed. Any change in the number of microglial cells in the hippocampus depending on the distance from the implanted cannula (1,200 μm in the caudal direction) was also assessed.

**Biochemistry**

The right hemisphere was placed in an ice-cold buffer containing 220 mM mannitol, 70 mM sucrose, 10 mM Hepes, and 1 mM EGTA (pH 7.35) immediately after rapid extraction from the skull (20–40 s). The brain tissue was cut into small pieces and placed in a homogenizer (Duran, Wheaton) with 4 ml of the buffer and homogenized manually for 1.5 min. The brain homogenate was fractionated by centrifugation at different rates. The homogenate was centrifuged at 4,000 *g *for 10 min at 4°C. The pellet was resuspended in 3 ml of a potassium phosphate buffer containing 125 mM KCl and 8 mM KH_2_PO_4_ (pH 7.4) to obtain a membrane-enriched fraction 1. The supernatant was centrifuged at 12,000 *g *for 15 min at 4°C. The cytosol-enriched supernatant constituted fraction 2. The resulting pellet was resuspended in 0.5 ml of the EGTA-free isolation medium to obtain mitochondria- enriched fraction 3. All the fractions were kept on ice during the experiment. Oxidative stress markers were determined in the three fractions obtained; hereinafter they are referred to as the membrane, cytoplasmic, and mitochondrial fractions. Evaluations were performed for half an hour using a CLARIOstar microplate reader (BMGlabtech, Germany).

Changes in reactive oxygen species (ROS) formation were determined by fluorimetry using the Amplex Red dye (30 μM, Thermo Fisher Scientific). The concentration of reduced sulfhydryl groups was measured using the Ellman photometric method with 1.3 mM 5,5’-dithiobis-( 2-nitrobenzoic acid) (DTNB, ThermoFisher, USA). The optical density was measured at a wavelength of 415 nm using the cysteine calibration curve. Peroxidized lipids were determined by fluorimetry of the products of the reaction with 7.5 mM thiobarbituric acid (TBARS assay, Sigma-Aldrich, USA). Fluorescence was measured with excitation at 530 nm, absorption at 554 nm, and a calibration curve of 1,1,3,3-tetraethoxypropane (Sigma-Aldrich, St. Louis, MO, USA). The protein content in fractions for biochemical normalization was determined using the Bradford method and Coomassie Brilliant Blue R-250. The optical density was measured at 595 nm using a calibration curve of bovine serum albumin. Since the cytoplasmic fraction had a higher protein content compared to the membrane and mitochondrial fractions, it had lower normalized values.

**Statistical analysis**

The statistical analysis was performed using the Mann–Whitney criterion and single-factor ANOVA method. Data were presented as mean ± SD,* p *≤ 0.05 (*), *p *≤ 0.01 (**), *p *≤ 0.001 (***).

## RESULTS

The experiments were performed in four animal groups: 1 – A control group that received a single dose of ACSF through a Hamilton syringe during neurosurgery (group C, *n *= 6); 2 – A group that received a single injection of Aβ1–42 through a Hamilton syringe during neurosurgery (group AD, *n *= 6); 3 – A control group with DMSO administration through an implanted cannula for 3 days (group C-C, *n *= 6); and 4 – animals that received a single injection of Aβ1–42 with subsequent administration of DMSO through an implanted cannula for 3 days (group AD-C, *n *= 7).

**Behavioral Open Field test**

**Fig. 2:**
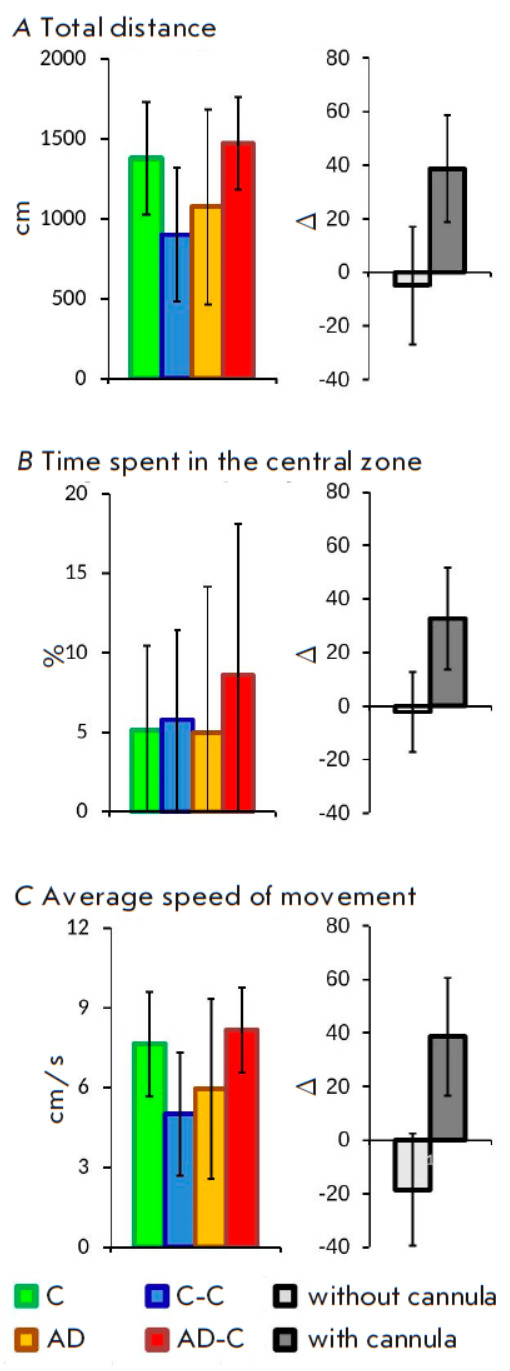
Behavioral analysis in experimental animals using the Open Field test. (*A*) Total distance traveled. (*B*) Total time spent in the central zone. (*C*) Average speed of movement. Data for each group of experimental animals are presented on the left; pairwise comparisons of control groups and groups receiving Aβ1-42 (C/AD and C-C/AD-C) are presented on the right. Data are presented as mean values + standard deviation

To assess the general condition of the mice, their motor activity and anxiety level in the new environment – the open field test – was carried out. The analysis of three parameters (distance traveled, speed of movement, and time spent in the central square) did not reveal any significant differences between the four study groups (*Fig. 2A,B,C*). Evaluation of relative changes between the two groups without cannulas (C and AD) and the two groups with cannulas (C-C and AD-C) showed that the presence of an implanted cannula changes the direction of behavioral reactions in the animals (*Fig. 2A,B,C*).

**Hippocampal microglia**

**Fig. 3:**
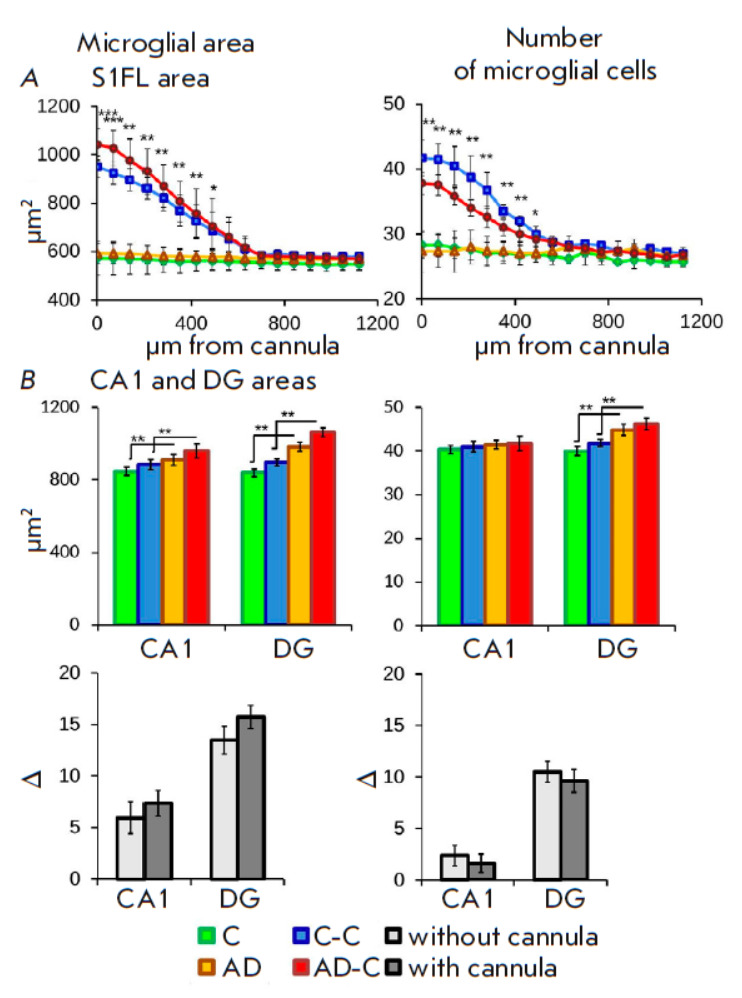
Immunohistochemical analysis of microglial cells. (*A*) Changes in the number and area size of microglial cells at a distance from the cannula implantation site to the occipital part of the brain. (*B*) Effect of cannulation and Aβ1-42 administration on microglia in the CA1 and DG fields of the hippocampus. Data for each group of experimental animals are presented at the top; pairwise comparisons of control groups and groups receiving Aβ1-42 (C/AD and C-C/AD-C) are presented at the bottom. Data are presented as mean values + stand - ard deviation. Significance: *p *≤ 0.05 (*), *p *≤ 0.01 (**),* p *≤ 0.001 (***)

Since any intervention in the brain leads to tissue damage to some extent and, as a result, microglia activation, the first goal was to evaluate the damage to the area around the implanted guide cannula by analyzing microglia. Two weeks after neurosurgery, sequential analysis of sections with increasing distance from the cannula was performed. Microglia activation caused by cannula implantation was noted at a distance of ~600 μm from the cannula (*Fig. 3A*). Cannula implantation increased the total area of microglial cells by 423.7 ± 74.1 μm2 in the control animals and by 449.9 ± 85.5 μm2 in AD animals. Meanwhile, the total cell number was increased by 13.5 ± 4.6 and 10.5 ± 3.8 in the control and AD animals, respectively.

Immunohistochemical analysis of microglia in the CA1 field and the hilus of the hippocampal DG revealed an increase in the cell area in the mouse AD model compared to the control group: by 63.3 ± 28.4 and 143 ± 42.4 μm2 in the animals without a cannula (AD-C) and by 143.1 ± 37.9 and 167.2 ± 29.6 μm2 in those with a cannula (AD-C – C-C), respectively. The cell number was increased only in the DG region (*Fig. 3B*).

A comparative pairwise analysis of the animals with and without cannula implantation revealed no significant differences between the groups in neither cell area nor cell number (*Fig. 3B*). This data indicates that implantation has no negative effect on microglial cell parameters (number and area) at a distance of >600 μm from the cannula.

**Biochemistry**

We performed a biochemical analysis of oxidative stress markers in the brain’s right hemisphere (contralateral to the cannula implantation site) in the experimental animals.

**Peroxidized lipids**

**Fig. 4:**
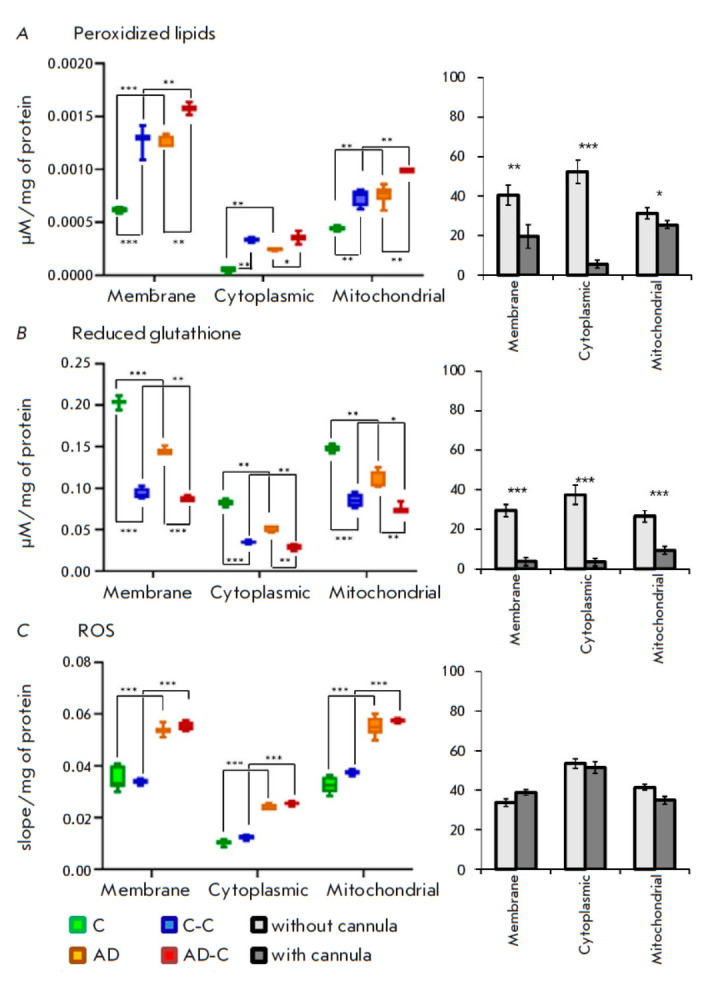
Biochemical analysis for oxidative stress markers. (*A*) Level of peroxidized lipids. (*B*) Reduced glutathione level. (*C*) ROS level. Data for each group of experimental animals are presented on the left; pairwise comparisons of the control groups and groups receiving Aβ1-42 (C/AD and C-C/AD-C) are presented on the right. Data are presented as mean values + standard deviation. Significance: *p *≤ 0.05 (*),* p *≤ 0.01 (**), *p *≤ 0.001 (***)

Analysis of the level of peroxidized lipids in the brain homogenate from the experimental animals demonstrated that cannula implantation results in a more than twofold increase in the level of peroxidized lipids in the first fraction, which mostly contains cell membranes (comparison of groups C and C-C, *p* < 0.01, *Fig. 4A*, *Table 1*). The fact that the cannula was implanted in the left half of the brain while the biochemical parameters were analyzed in the homogenate of the right half of the brain indicates that there was a significant effect of the damaged tissue on the overall level of peroxidized lipids in the brain.

**Table 1:** Level of oxidative stress markers in the brain homogenate fractions from experimental animals. Values are presented in % relative to the membrane fraction of the C-group and standard deviations

Oxidative stress marker	Fraction	C	C-C	AD	AD-C
Lipid peroxides	membrane	100 ± 4	204 ± 27	202 ± 9	253 ± 14
	cytoplasmic	9 ± 5	54 ± 4	39 ± 2	57 ± 14
	mitochondrial	71 ± 4	119 ± 13	121 ± 14	159 ± 1
Glutathione	membrane	100 ± 3	46 ± 3	70 ± 2	45 ± 1
	cytoplasmic	41 ± 2	17 ± 1	25 ± 2	16 ± 1
	mitochondrial	73±2	42 ± 4	54 ± 5	35 ± 1
ROS	membrane	100 ± 12	97 ± 3	154± 5	159 ± 5
	cytoplasmic	30 ± 3	36 ± 3	69 ± 3	73 ±2
	mitochondrial	91 ± 9	108 ± 3	159 ± 11	165 ± 3

In the animals without cannula implantation, Aβ administration resulted in a significant increase in the level of peroxidized lipids. In the first brain homogenate fraction, the level of peroxidized lipids was 6 × 10^4^ ± 0.2 × 10^4^ and 13 × 10^4^ ± 2 × 10^4^ μM/mg of protein in the C and AD groups, respectively. Amyloid injection through a cannula (AD-C group) resulted in an additional increase in the level of peroxidized lipids to 16 × 10^4^ ± 0.9 × 10^4^ μM/mg of protein. This dependence was observed in all three fractions (*Fig. 4A*). Furthermore, the level of peroxidized lipids in the C-C group was similar to that in the AD group (13 × 10^4^ ± 2 × 10^4^ and 12.6 × 10^4^ ± 0.5 × 10^4^ μM/mg of protein in the first fraction, respectively). This is indication of a chronic increase in free-radical oxidation in the contralateral hemisphere of the brain relative to the implantation site in the experimental animals.

Pairwise comparison of the groups with and without a cannula (*Fig. 4A*) showed Aβ administration resulting in a twofold increase of the oxidized lipids level in the first fraction in the animals without a cannula (comparison between the C and AD groups) compared to the animals with an implanted cannula (comparison between the C-C and AD-C groups).

**Glutathione**

Glutathione, an important factor determining the cell Red/Ox potential, is mainly located in the cytosol. Analysis of the glutathione level in a brain homogenate from the experimental animals showed that cannula implantation leads to a 2.4-fold decrease in the glutathione level in the second fraction, which mainly contains cytosol (comparison of groups C and C-C, *p* < 0.01, *Fig. 4B*, *Table 1*). This dependence was observed in all three fractions. This fact is indication of a significant impact of the implanted cannula on the overall glutathione level in the brain.

Amyloid administration resulted in a decrease in glutathione levels in all experiments. However, the changes in the animals without a cannula were 75% more pronounced compared to those in the animals with a cannula (AD/C groups – 40% and AD-C/C-C groups – 5%, *Fig. 4B*).

Interestingly, the glutathione level in the C-C group was lower than that in the AD group (0.035 ± 0.002 and 0.052 ± 0.004 μM/mg of protein in fraction 2, respectively). This indicates a chronic decrease in the regenerative capacity of the neural tissue due to the presence of the cannula in the brain of the experimental animals.

**Reactive oxygen species**

Cannula implantation did not affect the rate of ROS production: the values did not differ significantly between the C and C-C groups (*Fig. 4B*, *Table 1*).

Amyloid administration resulted in a similar increase of approximately 75% in the ROS production rate in all three homogenate fractions, regardless of the presence of a cannula (*Fig. 4B*, *Table 1*). Pairwise comparison of the groups (with and without a cannula) revealed no significant differences between them (*Fig. 4B*).

## DISCUSSION

In this work, we endeavored to study the effect of the guide cannula on the state of brain neural tissue in a mouse sAD model. Animal behavior was assessed at the systemic level; a immunohistochemical analysis of microglia and a biochemical analysis of brain oxidative stress markers were performed to assess the cellular and subcellular aspects of the impact. The analysis was undertaken two weeks after neurosurgery and Aβ1–42 administration, since an acute response to damage would have receded by this time, and changes in the parameters can be considered as signs of the initial stage of the chronic pathology.

Behavioral studies in an AD animal model, especially at early stages of the disease, are of interest, since they can be used to identify such psychoneurological disorders as hyperexcitability and anxiety. It has been shown that psychoneurological symptoms are an early manifestation of cognitive impairment in humans [30]. However, due to the fact that it is impossible to establish the moment of disease onset in humans, the specific stage of the onset of neurodegeneration in psychoneurological disorders remains unknown. In this regard, animal behavioral tests at different stages of AD are of particular interest. In the present study, the experimental animals were tested using the Open Field test. The Open Field test did not reveal substantive differences between the experimental groups two weeks after Aβ1–42 injection. However, pairwise comparison of the animals with and without a cannula showed that the implanted cannula changes the direction of the behavioral responses. A tendency towards decreased/ increased motor activity was noted in mice without/ with implanted cannula, respectively. This suggests a need to develop new behavioral tests that can detect early psychoneurological disorders in experimental AD model animals.

Since implantation of the guide cannula results in mechanical damage to brain tissue, it is important to distinguish between the effect of this damage and that of drugs injected chronically through the cannula. To achieve that, it is important to determine the area of mechanical damage around the cannula. Activated microglial cells, i.e. brain-resident macrophages, are one of the most commonly used biomarkers of neural tissue damage [31]. In our study, a comparative analysis of the parameters of the microglial cells and neural tissue around the cannula using a thin Hamilton syringe for injections showed that the number of activated microglial cells and their total area around the cannula increased by an average of 30 and 40%, respectively. The number of activated microglia gradually decreased with an increase in the distance from the cannula and reached the control level at a distance of 560 μm. For this reason, the effect of amyloid on hippocampal microglia in the animals with implanted cannulas was analyzed at a distance of >600 μm from the implantation site. In contrast to the use of a cannula, a single injection using a 0.33-mm Hamilton syringe had not increase the number and area of microglial cells two weeks after stereotactic surgery.

Administration of Aβ1–42 to animals treated both with and without the use of a cannula resulted in the activation of hippocampal microglia, which manifested itself in an increased cell area. Interestingly, the increase in the DG cell area was accompanied by growth in the cell number. At the same time, in the CA1 field, the number of microglial cells was unchanged. A comparative pairwise analysis of hippocampal microglia in the animals with and without a cannula showed that, although the level of microglia activation in the animals with a cannula was slightly higher, the presence of a cannula did not fundamentally affect the effect of Aβ1–42. Thus, our data demonstrate that local damage to neural tissue caused by a cannula implantation does not alter the effect of Aβ on the hippocampal tissues located at a sufficient distance (600 μm) from the cannula.

Since numerous studies have shown that oligomeric Aβ induces oxidative stress in neural tissue [32, 33], we analyzed oxidative stress markers in the right hemisphere (contralateral to the cannula implantation site) in the experimental animals. A comparative analysis of the two control groups (C and C-C) showed that cannula implantation had significantly increased the level of peroxidized lipids and drastically decreased the glutathione level in all three fractions (see *Table 1*). This suggests the development of chronic oxidative stress because of cannula implantation. At the same time, the ROS level did not differ between the groups.

The published data indicate that the decrease in glutathione level after cannula implantation may be associated with increased glutamate expression and excitotoxicity, which lead to enhanced oxidative stress, microglia activation, and zinc release, resulting in neuronal death. This manifests itself on the second day after mechanical injury to the neural tissue [34]. In addition, reactive activation of astrocytes takes place upon injury; these cells act as the main source of restored glutathione in the brain, which also results in a decreased glutathione level [35].

Amyloid administration in control animals (the AD group) led to an increased level of peroxidized lipids (by 100% in the membrane fraction) to the values in the C-C group. The decrease in glutathione level was significant (a 40% change in the cytoplasmic fraction when comparing the C and AD groups) but less pronounced than that caused by cannula implantation (a 60% change in the cytoplasmic fraction when comparing the C and C-C groups). The total ROS level changed by the same amount in the groups with and without implanted cannula.

Pairwise comparison of groups with cannula (C-C and AD-C) and without cannula (C and AD) demonstrated that cannula implantation, by triggering oxidative stress through mechanical damage to tissue, fundamentally reduces the severity of the effect of Aβ on peroxidized lipids and glutathione levels (twoand 10-fold, respectively). At the same time, it does not affect the ROS production rate. These data show that the total ROS level is an adequate and reliable proxy of disease development. At the same time, cannula implantation fundamentally distorts the effect of Aβ on the glutathione level in neural tissue, which can lead to false conclusions when interpreting experimental data on the mechanisms of sAD development and testing potential therapeutic drugs.

The data obtained here indicate that it is important to take into account the effect of the mechanical damage to tissue caused by an implanted cannula when analyzing the biochemical parameters of oxidative stress, which are widely used to assess the severity of experimental Alzheimer’s disease-type pathology.

## CONCLUSION

The study of the development the amyloid pathology with intracerebral administration of Aβ to BALB/c mice showed that the damage to neural tissue caused by a cannula implantation does not affect the behavioral and histological aspects of the Aβ effect. However, cannula implantation had a fundamental impact on (masked) the severity of the Aβ effect on the peroxidized lipids and glutathione levels in neural tissue. The ROS production rate did not depend on the presence of the cannula, thus confirming that this parameter is an adequate and reliable marker of pathology development. These facts indicate that cannula implantation unequally affects the biochemical markers of oxidative stress in response to amyloid injection. This is especially important to take into account in animal studies of the neural tissue redox state.

## Abbreviations

Aβ: β-amyloid
DG: dentate gyrus
ROS: reactive oxygen species
AD: Alzheimer’s disease
sAD: sporadic Alzheimer’s disease
group C: control group
group C-C: control group with cannula implantation
group AD: group with modeled Alzheimer’s disease
group AD-C: group with modeled Alzheimer’s disease and cannula implantation
